# Unenhanced multidetector computed tomography findings in acute central pulmonary embolism

**DOI:** 10.1186/s12880-019-0364-y

**Published:** 2019-08-14

**Authors:** Chiao-Hsuan Chien, Fu-Chieh Shih, Chin-Yu Chen, Chia-Hui Chen, Wan-Ling Wu, Chee-Wai Mak

**Affiliations:** 1 Department of Radiology, Chi-Mei Medical Center, No.901, Zhonghua Rd., Yongkang Dist, Tainan City, 710 Taiwan, Republic of China; 2 Graduate Institute of Medical Science, College of Health Science, Chang Jung Christian University, Tainan, Taiwan; 3 Department of Emergency, Chi-Mei Medical Center, No. 901, Zhonghua Rd., Yongkang Dist, Tainan City, 710 Taiwan, Republic of China

**Keywords:** CTPA, High attenuation, Pulmonary artery dilatation, Wedge-shaped consolidation

## Abstract

**Background:**

Computed tomography pulmonary angiography (CTPA) is the gold standard for the diagnosis of pulmonary embolism (PE). However, contrast is contraindicated in some patients. The purpose of this study was to determine the diagnostic accuracy of unenhanced multidetector CT (MDCT) for diagnosis of central PE using CTPA as the gold standard.

**Methods:**

The records of patients with suspected PE seen between 2010 and 2013 were retrospectively reviewed. Inclusion criteria were an acute, central PE confirmed by CTPA and non-enhanced MDCT before contrast injection. Patients with a PE ruled out by CTPA served as a control group. MDCT findings studied were high-attenuation emboli in pulmonary artery (PA), main PA dilatation > 33.2 mm, and peripheral wedge-shaped consolidation. Receiver operating characteristic (ROC) analysis was used to determine the sensitivity and specificity of unenhanced MDCT to detect PE. Wells score of all patients were calculated using data extracted from medical records prior to imaging analysis.

**Results:**

Thirty-two patients with a PE confirmed by CTPA and 32 with a PE ruled out by CTPA were included. Among the three main MDCT findings, high-attenuation emboli in the PA showed best diagnostic performance (Sensitivity 72.9%; Specificity 100%), followed by main PA dilatation > 33.2 mm (sensitivity 46.9%; specificity 90.6%), and peripheral wedge-shaped consolidation (sensitivity 43.8%; specificity 78.1%). Given any one or more positive findings on unenhanced MDCT, the sensitivity was 96.9% and specificity was 71.9% for a diagnosis of PE in patients. The area under the curve (AUC) of a composite measure of unenhanced MDCT findings (0.909) was significantly higher than that of the Wells score (0.688), indicating unenhanced MDCT was reliable for detecting PE than Wells score.

**Conclusions:**

Unenhanced MDCT is an alternative for the diagnosis of acute central PE when CTPA is not available.

## Background

Acute pulmonary embolism (PE) has an annual incidence of approximately 3–6 cases per 10,000 persons in the general population [1, 2], and is the third leading cause of death responsible for an average of 650,000 deaths annually in the United States [3–5].

Currently, the diagnostic strategy of PE mainly evaluates the hemodynamic status first, followed by clinical risk assessment system (Wells score and Geneva score). After confirmation of PE or ruling out non-PE patients using hemodynamic and clinical risk assessment test, the suspected PE patients may perform radiological assessment using multi-detector contrast-enhanced computed tomography angiography (CTPA), which is the gold standard for the imaging diagnosis of PE [6–10]. However, excessive use of CTPA may result in excessive radiation exposure. Furthermore, even though Wells score and revised Geneva score can rule-out non-PE patients [11–16], it cannot be used for definitive PE diagnosis [17]. The Wells score-revised Geneva score stratification method can be further combined with the D-dimer test [18], which is a useful, non-invasive approach for the diagnosis of PE. The predictive value of the D-dimer test depends greatly on the clinical pretest probability estimated by the Wells score [2, 6, 7, 19–22].

The use of contrast agents is contraindicated in certain patients, such as those with renal insufficiency [23]. Generally, physicians faced with this clinical situation will have the Wells score available for ruling out the PE, but evaluation tools or tests for detecting PE are lacking. Rapid diagnosis of PE has been shown to reduce the mortality rate [6], and waiting for laboratory tests of renal function before performing CTPA may delay diagnosis. Unenhanced multidetector CT (MDCT) might be used as alternative methods to get images as soon as possible. An acute PE can occasionally be detected as high-attenuation emboli in the pulmonary artery (PA) on unenhanced CT [24, 25]. Furthermore, acute central PE is associated with more severe hemodynamic changes and higher mortality than distal PE and chronic PE, and timely intervention is vital in achieving good treatment outcomes [26]. The ability of the radiologists to establish an accurate diagnosis of PE base on MDCT information may be helpful in a situation where CTPA cannot be performed or is not available. Only a few reports have addressed the utility of non-contrast CT images in PE detection focusing on high attenuation emboli found in PA [24, 27–29]. None of the previous studies have attempted to evaluate the diagnostic performance of multiple unenhanced MDCT findings or determine the most sensitive criteria for determining a diagnosis based on multiple unenhanced MDCT findings.

The purpose of this study was to determine the sensitivity and specificity of unenhanced MDCT for the diagnosis of PE using CTPA as the gold standard. We also sought to determine what unenhanced MDCT findings are most useful for diagnosis of PE, and compare the accuracy of MDCT and Wells score for diagnosing PE, again using CTPA as the gold standard. Our hypothesis was that unenhanced MDCT may present as an alternative approach for diagnosis of PE when CTPA is not available.

## Methods

### Patients

The study protocol was approved by the institutional review board of Chi-Mei Medical Center, and informed consent was waived based on the retrospective nature of this study.

The medical records of all patients who were admitted to the emergency department of our medical center with suspected PE between 2010 and 2013 were retrospectively reviewed. Acute central PE was the focus of this study because it is associated with more severe hemodynamic changes and higher mortality, and requires prompt intervention to have a good outcome as compared to distal PE and chronic PE [26]. Acute central PE was defined as a clot in the main, left, or right PA. Patients with a chronic PE were excluded. Chronic PE was defined as complete obstruction with an eccentric of calcified thrombus; post-stenotic dilatation of pulmonary artery, peripheral PA affected segments may be narrowed; PA calcification; right ventricular enlargement or hypertrophy is seen and lung mosaic perfusion pattern is present [30]. Patients who did not receive a CTPA or MDCT, and those without sufficient data in the medical records to calculate a Wells score were also excluded.

We have a CTPA protocol at our hospital. When a CTPA is considered necessary, first a non-enhanced CT is performed, followed by CTPA, and finally by a venous phase contrast enhanced CT. The case group consisted of patients with an acute, central PE confirmed by CTPA, who had also undergone non-enhanced MDCT of the chest. A control group with no evidence of PE confirmed by CTPA was randomly selected from the same time period. Records were first identified by ICD-9 code (415.1; pulmonary embolism and infarction includes acute and chronic, central and peripheral pulmonary embolism), and the images of those records identified were reviewed by radiologists on a picture archiving and communications system (PACS) workstation for identification of patients with an acute, central PE confirmed by CTPA.

### Imaging analysis

All imaging studies were performed on a Toshiba Aquilion 64 Slice CT, and the scanning protocol at the time included both unenhanced and enhanced scans. Both images were collected for evaluation. Parameters varied among the unenhanced and enhanced examinations, with a slice thickness ranging from 3 to 5 mm. All MDCT images were reviewed by two experienced radiologists (with 3 and 15 years of experience in reading CTPA, respectively) who were blinded to the patients’ medical history and examination and laboratory findings. The radiologists reviewed the records independently. When their independent observations did not agree, they attempted to achieve a consensus. If no consensus was achieved the patient was excluded. Only non-contrast images were reviewed by the radiologists to avoid possible misleading due to contrast-enhanced results. Three important radiologic features on unenhanced MDCT images were chosen to compare with the Wells score: High-attenuation emboli in pulmonary artery (PA), main PA dilatation > 33.2 mm, and peripheral wedge-shaped consolidation. Again, only when all features were agreed upon by the two radiologists was a patient included in the study.

### Wells score

Wells score was calculated based on seven variables are previously described [20]. The variables and their score were: 1) clinical symptoms of deep venous thrombosis (DVT) (score = 3.0); 2) no alternative diagnosis (score = 3.0); 3), heart rate > 100 (score = 1.5); 4) immobilization or surgery in the previous 4 weeks (score = 1.5); 5) previous DVT/PE (score = 1.5); 6) hemoptysis (score = 1.0); 7) malignancy (score = 1.0). The scores of the seven variables were summed to determine the Wells score. PE was considered unlikely if the Wells score was < 4.5, and considered likely if the score was ≥4.5. The combination of a Wells score <  4.5 and a negative SimpliRED D-dimer result was considered to exclude a PE [20]. Data were extracted from the medical records. If data of any of the seven variables was not available, the patient was excluded.

### Statistical analysis

The gold standard for the diagnosis of PE was CTPA. Categorical data were expressed as numbers and percentages. Fisher’s exact test was performed to examine the associations of Wells score items and unenhanced MDCT image findings with PE. Logistic regression analyses were performed to examine the associations of a diagnosis of PE based on CTPA with Wells score and the number of findings on unenhanced MDCT, as well as with each item of unenhanced MDCT. In order to select significant individual features that might help detect PE and to examine whether unenhanced MDCT findings were independently associated with PE diagnosis, multivariate logistic regression was performed by including both Wells score and number of MDCT findings, age, and sex of patients. The number of positive findings on unenhanced MDCT was considered as a continuous variable, and was included as one independent variable in the logistic regression model with PE diagnosis as the dependent variable. Odds ratios (ORs) were obtained from logistic regression. Receiver operating characteristic (ROC) curve analysis was performed to evaluate the diagnostic performance of unenhanced MDCT in detecting PE. The area under the ROC curve (AUC) for unenhanced MDCT was compared with that of the Wells score by using the method proposed by DeLong et al. [31]. Sensitivity, specificity, positive likelihood ratio (PLR), positive predictive value (PPV), negative likelihood ratio (NLR), and negative predictive value (NPV), as well as their 95% confidence intervals (95% CIs), for diagnosis of PE were calculated for each unenhanced MDCT finding. ROC curve analyses were performed by using MedCalc for Windows, version 12.5 (MedCalc Software, Ostend, Belgium). Descriptive statistics and regression analysis were performed by IBM SPSS statistical software version 22 for Windows (IBM Corp., New York, USA). A value of *p* <  0.05 was considered statistically significant.

## Results

A total of 181 patients were diagnosed with a PE during the study period. After applying the exclusion criteria, 32 patients with an acute central PE confirmed by CTPA, and 32 patients with a PE ruled-out by CTPA were included in the analysis. There was no significant difference between ages of individuals with and without a PE (mean age: 67.1 ± 16.6 versus 65.3 ± 14.6 years, respectively, *p* = 0.66). Approximately 41% of the patients with a PE were male, while 46.9% of those without a PE were male (*p* = 0.80).

**Fig. 1:**
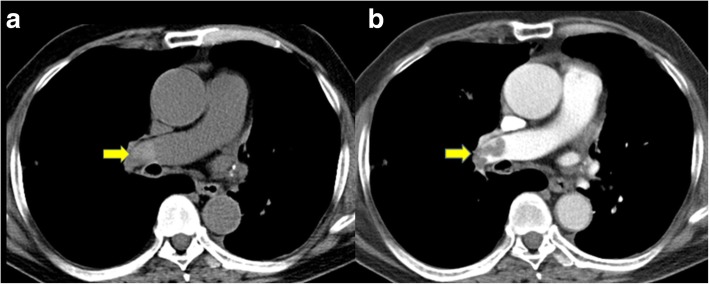
This patient was seen in the emergency department with dyspnea and diagnosed with an acute pulmonary embolism by CTPA. **a** Non-contrast computed tomography showed high attenuation emboli in the right pulmonary artery (arrow). **b** Post-contrast image showed filling defects in the right pulmonary artery

**Fig. 2:**
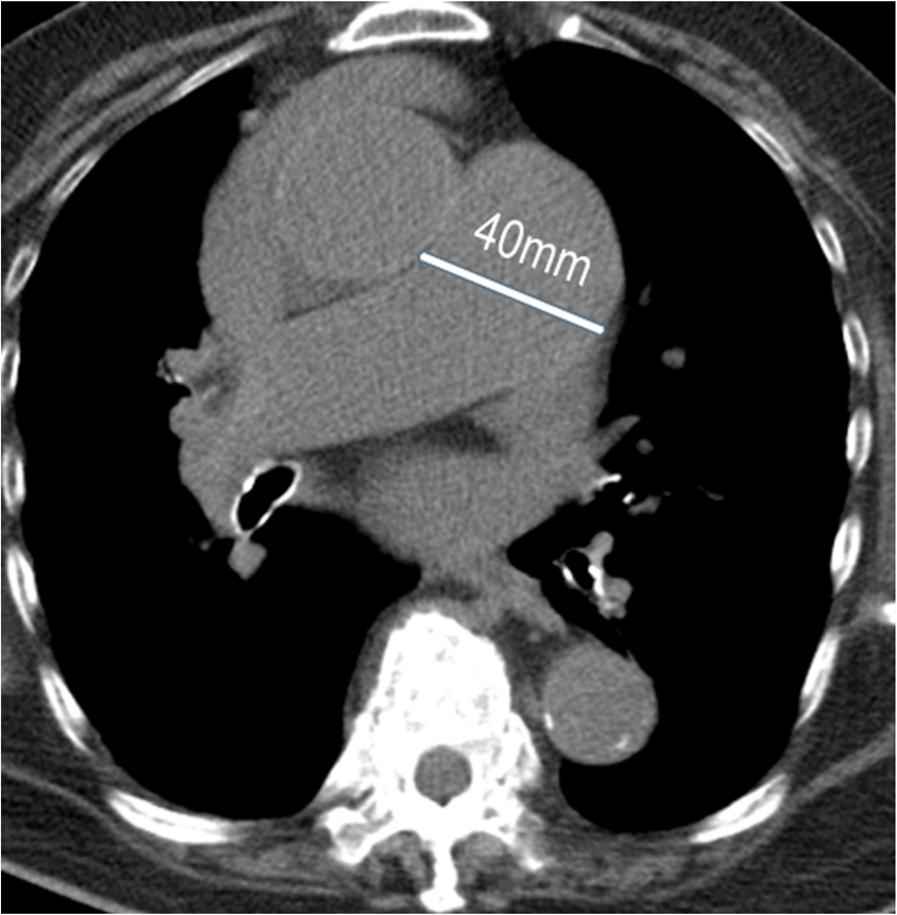
This patient was seen in the emergency department for dyspnea. Computed tomography showed a dilated pulmonary artery (diameter > 33.2 mm)

**Fig. 3:**
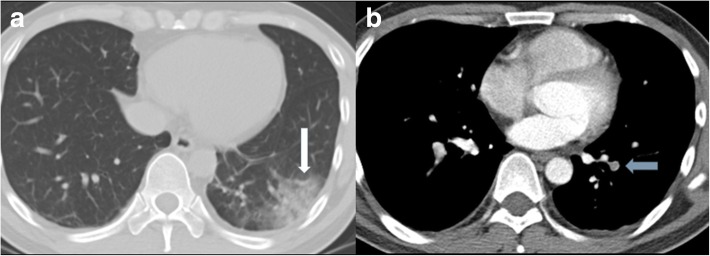
This patient was seen in the emergency department due to hemoptysis, and was diagnosed with an acute pulmonary embolism by CTPA. **a** A wedged-shaped opacification was seen in the left lower lobe (arrow). **b** Post-contrast image showed a centrally located embolism surrounded by contrast material (polo mint sign, arrow)

Wells criteria and unenhanced MDCT findings in patients with and without a PE are shown in Table 1. Based on Wells score, all 64 patients had an alternative diagnosis that was less likely than a PE. Approximately 75% of patients with a PE had a Wells score > 4.5, while 56.3% of patients without a PE had a Wells score > 4.5. The primary findings on unenhanced MDCT of the chest in patients with a PE were high attenuation within the pulmonary artery (35.9%), main PA dilatation > 3.2 mm (28.1%), and peripheral wedge-shaped consolidation (43.8%) (Table 1; Figs. 1, 2 and 3).

**Table 1:** Wells criteria and unenhanced multidetector computed tomography (MDCT) findings in patients with and without pulmonary embolism (PE)

	With PE diagnosed by CTPA	Without PE confirmed by CTPA	*p*-value
Wells criteria			
DVT	11 (34.4%)	0 (0%)	< 0.001
Alternative diagnosis less likely than PE	32 (100%)	32 (100%)	NA
Heart rate > 100 beats/minute	19 (59.4%)	14 (43.8%)	0.317
Recent surgery or immobilization	3 (9.4%)	5 (15.6%)	0.708
Previous PE/DVT	3 (9.4%)	4 (12.5%)	1.000
Hemoptysis	2 (6.3%)	0 (0%)	0.492
Malignancy history	6 (18.8)	4 (12.5%)	0.732
Wells score			0.188
≥ 4.5	24 (75.5%)	18 (56.3%)	
< 4.5	8 (25.0%)	14 (43.8%)	
Unenhanced MDCT			
High attenuation in pulmonary artery (PA)	23 (71.9%)	0 (0%)	< 0.001
Main PA dilatation > 33.2 mm	15 (46.9%)	3 (9.4%)	0.002
Peripheral wedge-shape consolidation	14 (43.8%)	7 (21.9%)	0.109
Number of findings			< 0.001
0	1 (3.1%)	23 (71.9%)	
1	13 (40.6%)	8 (25.0%)	
2	15 (46.9%)	1 (3.1%)	
3	3 (9.4%)	0 (0%)	

*DVT* deep vein thrombosis, *MDCT* multidetector computed tomography, *PA* pulmonary artery, *PE* pulmonary embolism

NA: Not applicable since all patients had alternative diagnosis less likely than PE

To compare the association of individual findings on unenhanced MDCT with PE diagnosis, we performed multivariate regression analysis. After adjusting for age, gender, and number of unenhanced MDCT findings, multivariable analysis indicated that diagnosis of PE was only associated with a greater number of findings on unenhanced MDCT (adjusted odds ratio [aOR] = 26.34; 95% CI: 4.91, 141.29; *p* <  0.001), and main PA dilatation > 33.2 mm (aOR = 10.59; 95% CI: 2.39, 47.02; *p* = 0.002) (Table 2). No associations were found for any of the Wells criteria.

**Table 2:** Association between PE diagnosis by enhanced MDCT with Wells score and unenhanced MDCT findings

	OR (95% CI)	*p*-value	aOR (95% CI)	*p*-value
Wells score (≥ 4.5 vs. < 4.5) ^a^	1.68 (1.17, 2.41)	0.005	2.10 (0.99, 4.42)	0.052
Number of findings on unenhanced MDCT^b^	21.11 (4.91, 90.77)	< 0.001	26.34 (4.91, 141.29)	< 0.001
High attenuation in pulmonary artery (PA) ^b^	NA			
Main PA dilatation > 33.2 mm ^b^	8.53 (2.15, 33.79)	0.002	10.59 (2.39, 47.02)	0.002
Peripheral wedge-shape consolidation ^b^	2.78 (0.93, 8.27)	0.066	2.79 (0.84, 9.20)	0.093

*aOR* adjusted odds ratio, *MDCT* multidetector computed tomography, *PE* pulmonary embolism

NA: Not applicable since there were no non-PE patients for this finding

^a^The multivariate model included age, gender, and number of findings on unenhanced MDCT

^b^The multivariate model included age, gender, and Wells score

The performance of unenhanced MDCT for the diagnosis of PE is shown in Table 3. High-attenuation emboli in pulmonary artery had the highest sensitivity (71.9%; 95% CI: 53.3, 86.3%; AUC = 0.859) and specificity (100%; 95% CI: 89.1, 100%) for the diagnosis of PE, followed by a main PA dilatation > 33.2 mm (sensitivity = 46.9%; specificity = 90.6%; AUC = 0.687), and peripheral wedge-shaped consolidation (sensitivity = 43.8%; specificity = 78.1%; AUC = 0.609). The optimal cut-off point for the number of findings on unenhanced MDCT was ≥1. The sensitivity was 96.9% (95% CI: 83.8, 99.9%) and specificity was 71.9% (95% CI: 53.3, 86.3%) for a diagnosis of PE in patients when there was at least one positive finding on unenhanced MDCT (Table 3).

**Table 3:** Diagnostic performance based on unenhanced MDCT findings

Unenhanced MDCT	Sensitivity (%)	Specificity (%)	PPV (%)	NPV (%)	PLR	NLR	AUC
High attenuation emboli in PA	71.9 (53.3, 86.3)	100 (89.1, 100)	100 (85.2, 100)	78.0 (62.4, 89.4)	NE	0.28	0.859
Main PA dilatation > 33.2 mm	46.9 (29.1, 65.3)	90.6 (75.0, 98.0)	83.3 (58.6, 96.4)	63.0 (47.5, 76.8)	5.0 (3.4, 7.4)	0.6 (0.2, 1.8)	0.687
Peripheral wedge-shape consolidation	43.8 (26.4, 62.3)	78.1 (60.0, 90.7)	66.7 (43.0, 85.4)	58.1 (42.1, 73.0)	2.0 (1.3, 3.1)	0.7 (0.3, 1.5)	0.609
Number of positive findings^a^	96.9 (83.8, 99.9)	71.9 (53.3, 86.3)	77.5 (61.5, 89.2)	95.8 (78.9, 99.9)	3.4 (2.7, 4.3)	0.04 (0.01, 0.3)	0.909

*AUC* area under ROC curve, *MDCT*, multidector computed tomography, *NE* not estimated, *PA* pulmonary artery, *PLR* positive likelihood ratio, *NLR* negative likelihood ratio, *PPV* positive predictive value, *NPV* negative predictive value

^a^The optimal cut-of-point was ≥1

**Fig. 4:**
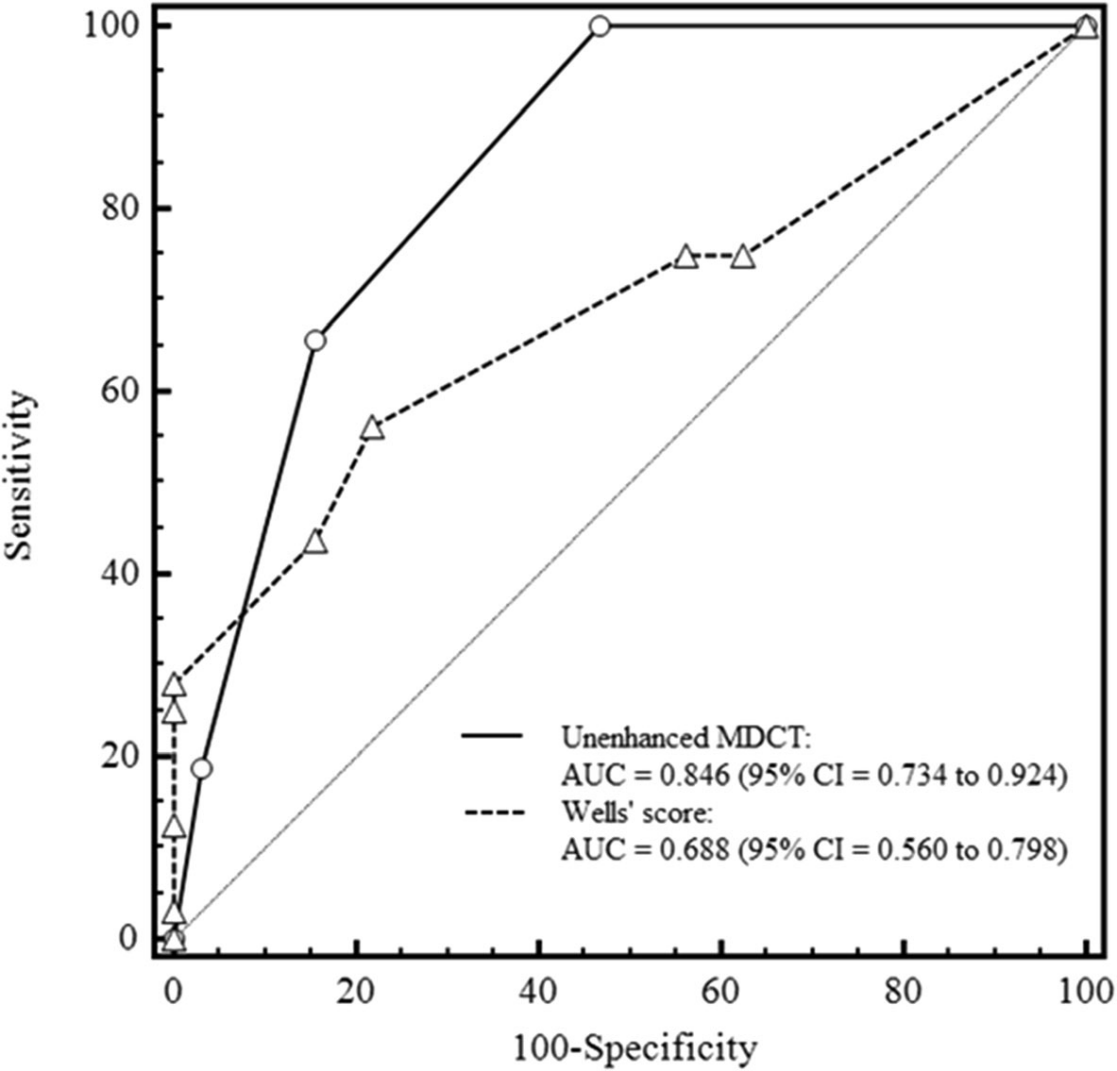
Receiver operating characteristic (ROC) curve for unenhanced MDCT and Wells score used for the diagnosis of pulmonary embolism

The AUC of a composite measure of unenhanced MDCT (i.e., number of findings on unenhanced MDCT) (AUC = 0.909; 95% CI: 0.811, 0.967) was significantly higher than that of the Wells score (AUC = 0.688; 95% CI: 0.560, 0.798) (*p* = 0.002), indicating better diagnostic performance of unenhanced MDCT than Wells score for detecting a PE (Fig. 4).

## Discussion

The purpose of this study was to determine the value of unenhanced MDCT as screening tool for central acute PE using CTPA as the gold standard. The sensitivity was 96.9% and specificity was 71.9% for a diagnosis of PE in patients with at least one positive finding on unenhanced MDCT. High attenuation within the PA had a PPV of 100% and NPV of 78.0% for diagnosis of a central acute PE. Furthermore, the diagnostic performance of unenhanced MDCT was significantly better than that of Wells score. These results suggest that unenhanced MDCT may be useful for a rapid diagnosis of PE when CTPA is not available or contraindicate.

Acute PE is a life-threatening condition and prompt diagnosis is critical for good outcomes. While CTPA is the gold standard for diagnosis, it is not always available and contraindicated in certain patients. Wells score is typically used as alternative, and while useful for ruling out PE it is not sensitive for diagnosis of a PE [17]. For this reason, we examined the value of an alternative, unenhanced MDCT, as a screening to for the diagnosis of PE in the emergency room setting. The finding of high attenuation emboli in the PA on unenhanced MDCT has received most attention and evaluated by several studies in the context of PE diagnosis. Moreover, wedge-shaped subpleural consolidation and dilated central pulmonary arteries observed in unenhanced MDCT had been indicated as indirect signs for acute PE [32]. To the best of our knowledge, the current study is the first in performing and proposing a multi-component evaluation strategy based on several imaging findings on unenhanced MDCT. High attenuation emboli in PA indeed showed best diagnostic performance among the three analyzed findings in the present study, and the sensitivity was further improved by inclusion of other unenhanced MDCT findings. Further investigations performed in a more general setting or in a prospective manner are required for confirming the favorable diagnostic performance shown by multi-component unenhanced MDCT findings before advice on implementation of the strategy can be made.

Although we showed that high-attenuation emboli in PA had a sensitivity of 71.9% and specificity of 100% for diagnosis of a PE, other studies reported slightly lower sensitivity or incidence of the unenhanced MDCT finding. Tatco et al. [24] reported that this sign had an overall sensitivity of only 36% for detecting central PE, which is significantly lower that the sensitivity found in our study. Cobelli et al. [28] reported that emboli in central PA could be detected on unenhanced CT in 41.2% of their hospitalized patients with clinical suspicion of PE, and Kanne et al. [27] found that 46.1% of their unenhanced scans with central clots and 6% of all unenhanced CT scans carried out in their institution were positive for PE. High-attenuation emboli in PA had a higher sensitivity for PE in the current study, probably because we focused on acute emboli suspected in the emergency room, and the diagnosis was only made when there was consensus of two radiologists. The high attenuation of thrombi on CT is due to the higher level of hemoglobin in clots as compared to that of circulating blood [24, 33].

PA enlargement and wedge-shaped consolidation are well-known indicators suggestive of PE [34]. The use of PA enlargement and wedge-shaped consolidation in combination with high attenuation emboli in the PA is responsible for the overall high sensitivity of unenhanced MDCT for diagnosis of PE, and when at least one positive finding was noted on unenhanced CT the sensitivity approached 100%.

When emboli are located in segmental, subsegmental, and more peripheral arteries, the sensitivity of unenhanced CT is limited [35, 36]. Motion artifact, partial volume averaging, and low signal-to-noise ratio almost always affect imaging of the peripheral arteries and contribute to false negative results [36, 37]. In addition, any anatomical structure adjacent to the PA can cause areas of hyper-attenuation during respiratory or cardiac motion, which can mimic the hyperdense lumen sign. When volume averaging with atherosclerotic disease involving the pulmonary arteries is performed, false positive results may be obtained [24].

### Limitations

There are several limitations to this study, including its retrospective nature and the small sample size. All patients in this study were selected from the emergency department which may be one source of bias. The age of the clot and the patient’s hematocrit level at the time of imaging, and other factors which may interfere with visualization were not assessed. In addition, the study examined only acute central PE, and thus the technique may not be of value for imaging of other types of PE. High attenuation emboli in the PA were the greatest source of discrepancy between radiologists because of the non-quantitative and subjective nature of this finding, and we did not determine inter-rater accuracy of diagnosis. We acknowledge that the included patient numbers were not large, future studies with larger sample size is necessary for further validation. However, the results from this study does demonstrate that unenhanced MDCT is an alternative approach for the diagnosis of PE.

## Conclusions

Unenhanced MDCT is an alternative approach for the diagnosis of acute central PE when CTPA is inaccessible or contraindicated. In our study, non-enhanced MDCT has shown better performance than Well’s score for confirming acute thrombi in the main right or left pulmonary arteries, but cannot rule out pulmonary thromboembolism.

## Data Availability

All data generated or analysed during this study are included in this published article.

## Abbreviations

aOR: Adjusted OR
AUC: Area under the ROC curve
CTPA: Computerized tomography pulmonary angiography
DVT: Deep vein thrombosis
MDCT: Unenhanced multidetector computerized tomography
NPV: Negative predictive value
OR: Odds ratio
PE: Pulmonary embolism
PPV: Positive predictive value
ROC: Receiver operator characteristic
